# Cardiopulmonary Bypass Induces Acute Lung Injury via the High-Mobility Group Box 1/Toll-Like Receptor 4 Pathway

**DOI:** 10.1155/2020/8854700

**Published:** 2020-09-25

**Authors:** Yuxiao Deng, Lei Hou, Qiaoyi Xu, Qi Liu, Su Pan, Yuan Gao, Richard A. F. Dixon, Zhengyu He, Xiangrui Wang

**Affiliations:** ^1^Department of Critical Care Medicine, Renji Hospital, School of Medicine, Shanghai Jiaotong University, Shanghai 200127, China; ^2^Department of Anesthesiology and Critical Care, Shanghai East Hospital, Tongji University School of Medicine, Shanghai 200120, China; ^3^Texas Heart Institute, Houston, Texas, USA

## Abstract

During cardiopulmonary bypass (CPB), pulmonary ischemia/reperfusion (I/R) injury can cause acute lung injury (ALI). Our previous research confirmed that abnormal high-mobility group box 1 (HMGB1) release after CPB was closely related to ALI. However, the mechanism underlying the HMGB1-mediated induction of ALI after CPB is unclear. Our previous study found that HMGB1 binds Toll-like receptor 4 (TLR4), leading to lung injury, but direct evidence of a role for these proteins in the mechanism of CPB-induced lung injury has not been shown. We examined the effects of inhibiting HMGB1 or reducing TLR4 expression on CPB-induced lung injury in rats administered anti-HMBG1 antibody or TLR4 short-hairpin RNA (shTLR4), respectively. In these rat lungs, we studied the histologic changes and levels of interleukin- (IL-) 1*β*, tumour necrosis factor- (TNF-) *α*, HMGB1, and TLR4 after CPB. After CPB, the lung tissues from untreated rats showed histologic features of injury and significantly elevated levels of IL-1*β*, TNF-*α*, HMGB1, and TLR4. Treatment with anti-HMGB1 attenuated the CPB-induced morphological inflammatory response and protein levels of IL-1*β*, TNF-*α*, HMGB1, and TLR4 in the lung tissues and eventually alleviated the ALI after CPB. Treatment with shTLR4 attenuated the CPB-induced morphological inflammatory response and protein levels of IL-1*β*, TNF-*α*, and TLR4 in the lung tissues and eventually alleviated the ALI after CPB, but could not alleviate the HMGB1 protein levels induced by CPB. In summary, the present study demonstrated that the HMGB1/TLR4 pathway mediated the development of ALI induced by CPB.

## 1. Introduction

Cardiopulmonary bypass (CPB) performed during cardiac surgery is a well-known trigger of a robust inflammatory response that has been shown to contribute to dysfunction in several organs, including the lungs [1–3]. Numerous studies have suggested that a signalling pathway mediated by Toll-like receptor 4 (TLR4) plays a pivotal role in the pathogenesis of this inflammatory response in acute lung injury (ALI) [4, 5]. TLR4 levels have been shown to be significantly increased in the lung tissues after CPB [6]. Furthermore, in rats, nuclear factor- (NF-) *κ*B suppression induced through the inhibition of the upstream signalling molecules TLR4 and MyD88 ameliorated the pulmonary inflammatory response associated with CPB [6]. However, because of technical difficulties in performing CPB in TLR4 knockout mice, the in vivo study of TLR4 and its mechanistic role in CPB-induced lung injury has been limited.

TLR4 can be activated by pathogen-associated molecular patterns (PAMPs), such as lipopolysaccharide (LPS), and damage-associated molecular pattern (DAMP) molecules, such as high-mobility group box 1 (HMGB1). HMGB1, a DAMP protein, was reported to be involved in sterile inflammatory responses via the TLR4 signalling pathway [7]. In addition, our earlier research indicated that HMGB1 induced ALI through TLR4 in a dose-dependent manner [8]. Moreover, in rabbits with ventilator-induced lung injury, the damage could be attenuated by the intratracheal administration of an anti-HMGB1 antibody [9]. Although these studies suggest a potential role for the HMGB1/TLR4 pathway in lung injury, whether the HMGB1/TLR4 pathway is associated with CPB-induced ALI is unclear.

HMGB1 is not only a nonhistone nuclear protein [10] but has also been described as a proinflammatory cytokine involved in important organ processes, such as liver and brain injury [11, 12]. In addition, our team previously confirmed that HMGB1, as an important cytokine, plays an important role in mediating the acute damage and subsequent inflammatory processes in ALI [13, 14]. Other researchers have also found that pulmonary tissue damage could be alleviated by neutralizing HMGB1 with a specific antibody in rats [15], identifying HMGB1 as a therapeutic target for ALI.

In this study, we hypothesized that HMGB1-induced TLR4 signalling activation may be involved in the pathogenesis of the inflammatory responses in CPB-induced ALI.

## 2. Materials and Methods

### 2.1. Animals and Experimental Groups

Three-month-old male Sprague-Dawley rats (400–450 g; SLAC Laboratory Animal Company, Shanghai, China) were randomly assigned. Rats were housed under a controlled temperature (22–24°C) and a 12 h light/dark cycle (8:00–20:00 light; 20:00–8:00 dark) and had free access to food and tap water. All animals received humane care in accordance with the National Institutes of Health (NIH) Guide for the Care and Use of Laboratory Animals (Department of Health and Human Services, NIH Publication No. 86–23, revised 1985).

The rats were randomly divided into ten groups according to the needs of experiment (*n* = 6 per group):

- Control group: sham surgery
- CPB group: cardiopulmonary bypass
- Anti-HMGB1 group: anti-HMGB1 polyclonal antibody treatment one hour before sham surgery
- IgY group: IgY antibody treatment one hour before sham surgery
- Anti-HMGB1+CPB group: anti-HMGB1 polyclonal antibody treatment one hour before cardiopulmonary bypass
- IgY+CPB group: IgY antibody treatment one hour before cardiopulmonary bypass
- shTLR4 group: shTLR4 treatment (the rats were treated with TLR4-targeting short-hairpin RNA) three weeks before sham surgery
- shNT group: shNT treatment (the rats were treated with the nontargeting shRNA) three weeks before sham surgery
- shTLR4+CPB group: shTLR4 treatment three weeks before cardiopulmonary bypass
- shNT+CPB group: shNT treatment three weeks before cardiopulmonary bypass

Two hours after surgery, rats in each group were sacrificed and lung tissues were harvested for the next experiment.

### 2.2. Inhibition of TLR4

The inhibition of TLR4 expression was performed as previously reported [8]. Briefly, three weeks before CPB, the rats were intratracheally administered 5 × 10^7^ transducing units (TU) of lentivirus (shTLR4 or shNT) diluted with 0.25 mL of PBS. The sense strand insert sequence was 5′aaCCTAGAACATGTGGATCTT 3′. Biofluorescence imaging was performed to assess the lentiviral transduction rate via a NightOWL II 983 in vivo imaging system (Berthold Technologies, Bad Wildbad, Germany), and western blot analysis was used to verify the reduction of TLR4 expression in lung tissue.

### 2.3. HMGB1 Neutralization

The rats were injected intravenously with 2 mg/kg of chicken neutralizing anti-HMGB1 polyclonal antibody (Shino-Test Co., Kanagawa, Japan) or the same dose of chicken IgY antibody (Abcam Cambridge, UK) one hour before CPB according to the manufacturer's instructions. The initial mechanism of anti-HMGB1 antibody mainly depends on the neutralizing mAbs specifically binding to HMGB1 to inhibit its activity [16]. The neutralizing effect of anti-HMGB1 in the lungs were verified via enzyme-linked immunosorbent assays (ELISAs) and Western-Blot.

### 2.4. Surgical Procedures

Rats were anaesthetized with an intraperitoneal injection of 3% pentobarbital sodium (2 mL/kg) and were cannulated with a 16 G catheter (BD Insyte-W; BD Vialon Biomaterial, Franklin Lakes, NJ, USA) that was used as a tracheal tube. The rats were mechanically ventilated (TOPO Small Animal Ventilator; Kent Scientific, Torrington, CT, USA) with a 10 mL/kg tidal volume, 60 breaths/min respiratory rate, and a 100% inspiratory concentration of O_2_. The left femoral artery was dissected and cannulated with a 22 G catheter (BD Insyte-W) for continuous monitoring of the mean arterial pressure (MAP; Viridia 24C monitor; Hewlett-Packard, Palo Alto, CA, USA). The right carotid artery was dissected and cannulated with a 22 G catheter (BD Insyte-W), which was used for the arterial inflow in the CPB circuit. Before the onset of CPB, rats were administered heparin (250 IU/kg). The right external jugular vein was dissected and cannulated with a 14 G catheter (BD Insyte-W) that was modified to include multiple side orifices in the forepart. The 14 G catheter was inserted into the right jugular vein and advanced to the right atrium. This catheter was used for venous return to the CPB circuit. All incisions were performed after local injection of bupivacaine. The CPB circuit comprised a venous reservoir (a 5 mL cylinder syringe), a roller pump (Masterflex L/S; Cole-Parmer Instrument Co., Vernon Hills, IL, USA), and a specially designed membrane oxygenator (oxygenator with a surface area of 0.1 m^2^; Dongguan Kewei Medical Instrument Co., Ltd., Dongguan, China). The circuit was primed with 8 mL of hydroxyethyl starch (130/0.4), 0.5 mL of 5% NaHCO_3_, and 4 mL of lactated Ringer's solution. Throughout the experiment, the rectal temperature of each rat was monitored and maintained at 36.5°C to 38.5°C via a heating pad (ALC-HTP; Shanghai Alcott Biotech Co., Ltd., Shanghai, China). The blood flow rate was adjusted to the target CPB rate of 120 to 140 mL/kg/min and was maintained at this level for 1 h. During CPB, the MAP was sustained at 60 to 80 mmHg. When each rat was weaned off CPB, the right jugular vein was decannulated, and the remaining priming solution was infused into the bloodstream. After two hours of intensive postoperative care, the cannulas were removed, and the incisions were sutured. Rats in the control group underwent sham surgery.

### 2.5. Histopathology

The right upper lobe of the lung was collected from the rats after treatment with antibody or shRNA and two hours after the termination of CPB. The rats were euthanized with pentobarbital sodium (100 mg/kg), and their lungs were removed and fixed in 10% buffered formaldehyde solution overnight. The next day, the tissues were embedded in paraffin. Serial slices of each sample from the apex to the base were acquired, and 2 randomly selected sections (each 5 *μ*m thick) were stained with haematoxylin and eosin. Histologic sections were evaluated by a pathologist who was blinded to the experimental groups. Ten fields were randomly chosen from each section (a total of 20 fields per rat) and examined with an Olympus BX51 microscope (Olympus, Japan). A separate grade from 0 to 3 was calculated for each field by using the number of interstitial infiltrated neutrophils or the maximum width of the alveolar septa as an index of interstitial edema. The number of interstitial infiltrated neutrophils in each field was graded as follows: less than 5 = 0; 5–100 = 1; 100–200 = 2; and more than 200 = 3. The maximum width of the alveolar septa (actual size in mm) in each field was graded as follows: less than 5 = 0; 5–20 = 1; 20–50 = 2; and more than 50 = 3. For each variable, a single score was calculated as the sum of the field scores for each rat. A total lung injury score was calculated as the sum of the 2 components.

### 2.6. ELISA

Lungs were harvested and washed three times in PBS. The samples were then homogenized and centrifuged at 11, 000 × g at 4°C for 15 min, and the supernatant was collected. The tumour necrosis factor- (TNF-) *α* and interleukin- (IL-) 1*β* levels in the lungs were measured with commercial ELISA kits (R&D Systems, Minneapolis, MN, USA) according to the manufacturer's instructions. The HMGB1 levels in the lungs were measured with a commercial ELISA kit (Shino-Test Co., Kanagawa, Japan) according to the manufacturer's instructions.

### 2.7. Western Blot Analysis

Protein extraction and quantification were performed by using the same procedures described for the ELISA. For western blot analysis, 20 *μ*g of total protein per sample was loaded and run on a 10% sodium dodecyl sulphate (SDS) polyacrylamide gel. The proteins were then electrotransferred to nitrocellulose filter membranes. The membranes were incubated for two hours at 25°C in PBS containing 5% nonfat dry milk, followed by incubation for two hours at 25°C with primary antibodies against TLR4 (1 : 1000; Cell Signaling Technology, Danvers, MA, USA), HMGB1 (1 : 1000; Abcam, USA), or *β*-actin (1 : 2000; Bioworld Technology, St. Louis Park, MN, USA). The membranes were washed and then incubated with IRDye 800CW-conjugated goat anti-rabbit secondary antibody (1 : 5000; Santa Cruz Biotechnologies, CA, USA) for one hour at 25°C. Infrared fluorescence images were obtained with an Odyssey infrared imaging system (Li-Cor Biosciences, Lincoln, NE, USA), and band intensities were quantified with Image-Pro Plus 5.1 software (Media Cybernetics, Bethesda, MD, USA).

### 2.8. Statistical Analysis

All values are expressed as the mean ± standard deviation (SD). Groups were compared by using analysis of variance (ANOVA), followed by Tukey's multiple comparison. A two-sided probability value (*P*) less than 0.05 was considered significant.

## 3. Results

### 3.1. CPB Induced TLR4 and HMGB1 Expression in the Lung Tissues

**Figure 1:**
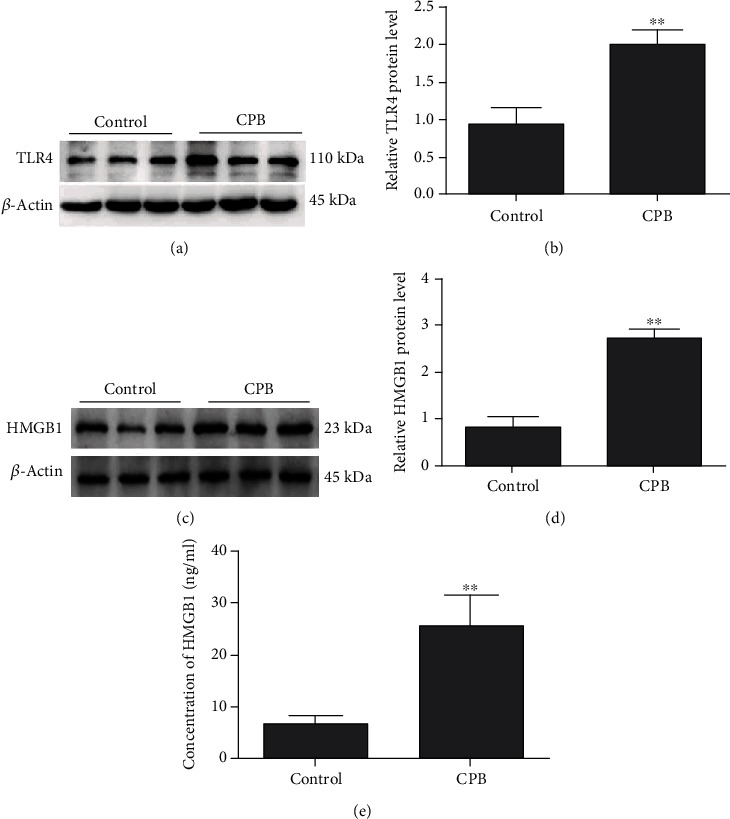
CPB induced TLR4 and HMGB1 expression in the lung tissues. (a) Western blot showing the TLR4 protein expression in the lung tissues of the control and CPB groups. (b) TLR4 protein density relative to that of *β*-actin. The TLR4 protein level was significantly higher in the CPB group than in the control group (a, b). (c) Western blot showing the HMGB1 protein expression of lung tissues in the control and CPB groups. (d) The HMGB1 protein density relative to that of *β*-actin. (e) The concentrations of HMGB1 were evaluated by ELISAs. HMGB1 protein level is much higher in the CPB group than in the control group by using western blot and ELISAs (c–e). Data are represented as the mean ± SD, *n* = 6 per group. ^∗∗^*P* < 0.01 vs. the control.

To investigate the TLR4 expression in lung tissues, we used western blot analysis to detect the TLR4 protein level in the lung tissues at two hours after the operation (*n* = 6). The TLR4 expression in the lung tissues was significantly increased in the CPB group compared with the control group (*P* < 0.01) (Figures 1(a) and 1(b)). To investigate the HMGB1 expression in lung tissues, we used western blot and ELISA to detect the HMGB1 protein level in the lung tissues at two hours after the operation (*n* = 6). The HMGB1 expression in the lung tissues was significantly increased in the CPB group compared with the control group (*P* < 0.01) (Figures 1(c)–1(e)).

### 3.2. Anti-HMGB1 Decreased CPB-Induced TLR4 Expression in the Lung Tissues

**Figure 2:**
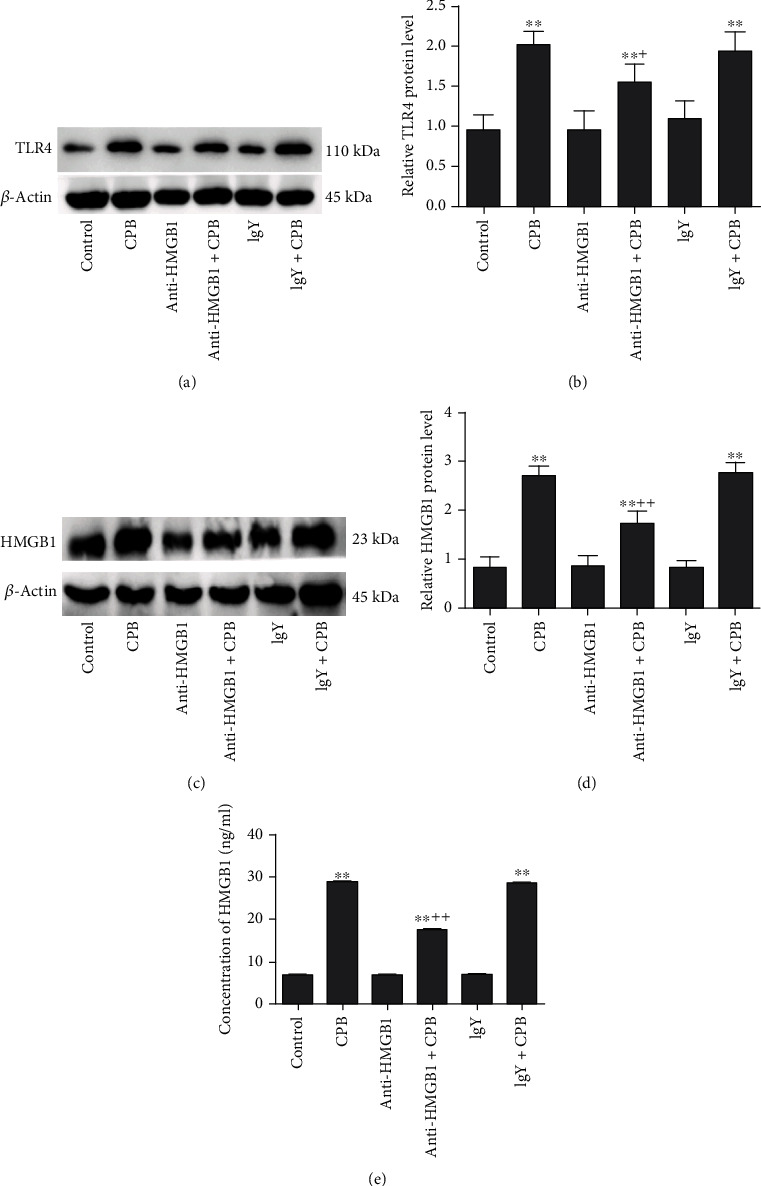
Anti-HMGB1 decreased CPB-induced TLR4 expression in the lung tissues. (a) Western blot results showing the TLR4 protein expression in the IgY+CPB, IgY, anti-HMGB1+CPB, anti-HMGB1, CPB, and control groups. (b) The TLR4 protein density relative to that of *β*-actin. The TLR4 protein level was significantly lower in the anti-HMGB1+CPB group than in the CPB and IgY+CPB groups (a, b). (c) Western blot showing the HMGB1 protein expression in the control, CPB, anti-HMGB1, anti-HMGB1+CPB, IgY, and IgY+CPB groups. (d) The HMGB1 protein density relative to that of *β*-actin. (e) The concentrations of HMGB1 were evaluated by ELISAs. The anti-HMGB1 mAb attenuated the CPB-induced increase in the HMGB1 levels by using western blot and ELISAs (c–e). Data are represented as the mean ± SD, *n* = 6 per group. ^∗^*P* < 0.05 and ^∗∗^*P* < 0.01 vs. control; ^+^*P* < 0.05 and ^++^*P* < 0.01 vs. CPB only.

To assess the relationship between HMGB1 and TLR4 in the lung tissues after CPB, we utilized an anti-HMGB1 antibody to block abnormal HMGB1 expression in the lung tissues after CPB and then examined the TLR4 protein expression by western blots (Figures 2(a) and 2(b)). No difference was observed among the control-, IgY-, or anti-HMGB1-treated groups in the absence of CPB. After CPB, the TLR4 protein expression was significantly increased (*P* < 0.01 vs. control) (Figures 2(a) and 2(b)). However, treatment of rats with the anti-HMGB1 neutralizing antibody significantly diminished the effect of CPB on the TLR4 protein expression in lung tissues (*P* < 0.05 vs. CPB only) (Figures 2(a) and 2(b)). Furthermore, we assessed the HMGB1 levels in the lung tissues after CPB by using the western blot and ELISA; after CPB, the HMGB1 protein expression was significantly increased (*P* < 0.01 vs. control) (Figures 2(c)–2(e)). Treatment of rats with the anti-HMGB1 neutralizing antibody significantly diminished the effect of CPB on the HMGB1 protein expression in lung tissues (*P* < 0.01 vs. CPB only) (Figures 2(c)–2(e)); its suppression efficiency reaches 40%.

### 3.3. Anti-HMGB1 Reduced CPB-Induced Acute Lung Injury in Rat Models

**Figure 3:**
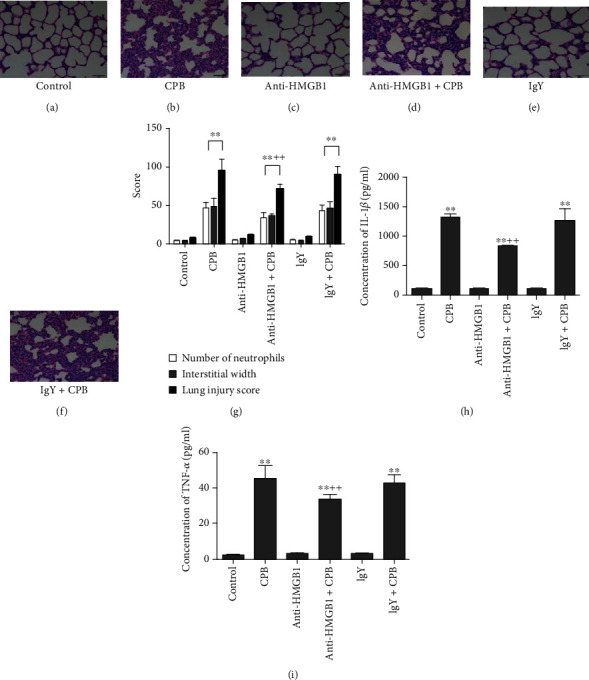
Anti-HMGB1 reduced CPB-induced acute lung injury in rat models. The histological changes of I/R lung injury induced by CPB were evaluated via H&E staining in comparisons of (a) control vs. (b) CPB, (c) anti-HMGB1 vs. (d) anti-HMGB1+CPB, and (e) IgY vs. (f) IgY+CPB. The number of neutrophils, interstitial width, and lung injury score were quantified and are shown in the different groups (g). Compared with the control group, the CPB-only and IgY+CPB groups showed histologic features of lung injury and increased lung injury scores. In the anti-HMGB1+CPB group, however, the lung injury was decreased, and the lung injury scores were lower than those in the CPB-only group. The concentrations of (h) IL-1*β* and (i) TNF-*α* were evaluated by ELISAs. The anti-HMGB1 mAb attenuated the CPB-induced increase in IL-1*β* and TNF-*α* levels of lung tissues. Data are represented as the mean ± SD, *n* = 6 per group. ^∗^*P* < 0.05 and ^∗∗^*P* < 0.01 vs. control; ^++^*P* < 0.01 vs. CPB only.

Our earlier research indicated that HMGB1 induced lung injury in a dose-dependent manner [8]. First, a CPB model (Figure 3(b)), indicated by alveolar septal thickening, interstitial edema, vascular congestion, and neutrophil infiltration in the interstitium, was established. To examine the potential role of HMGB1 in the pathogenesis of the lung injury induced by CPB, we treated CPB rats with an anti-HMGB1 antibody or IgY and histologically examined the lung tissues two hours after the termination of CPB. The rats that received anti-HMGB1 antibody (Figure 3(c)) or IgY only (Figure 3(e)) before sham surgery, and served as an appropriate control for each group of comparisons, showed no pathologic changes. We compared the anti-HMGB1+CPB and CPB-only groups and found that treatment with an anti-HMGB1 antibody significantly reduced the lung injury induced by CPB, as shown by the recovered alveolar septal thickening and vascular congestion in histological analysis (Figure 3(d)), as well as the reduced lung injury score, a quantitative evaluation of neutrophil infiltration and interstitial edema (Figure 3(g)). This pathological restoration was specific because the IgY+CPB and CPB-only groups showed no differences (Figures 3(f) and 3(g)). These results demonstrated that anti-HMGB1 attenuates the severity of the lung injuries after CPB. To assess the relationship between HMGB1 and other inflammatory cytokine in the lung tissues after CPB, we further examined the expression levels of the inflammatory cytokines IL-1*β* and TNF-*α* (Figure 3(i)) by using ELISAs. Consistent with the lung injury score data, the addition of anti-HMGB1 under CPB downregulated IL-1*β* and TNF-*α*, while the IgY+CPB and CPB-only groups showed similar levels of these inflammatory cytokines (Figures 3(h) and 3(i)). Taken together, our data showed that CPB triggered lung injury in rats, as demonstrated by the histologic changes and increased concentrations of various inflammatory cytokines and chemokines, including HMGB1, as well as the increased TLR4 expression in the lungs. Administration of anti-HMGB1, which had an inhibitory efficiency of 40% in the lungs, diminished the lung injury caused by CPB.

### 3.4. Knocking Down TLR4 Could Not Reduce CPB-Induced HMGB1 Protein Expression in the Lung Tissues

**Figure 4:**
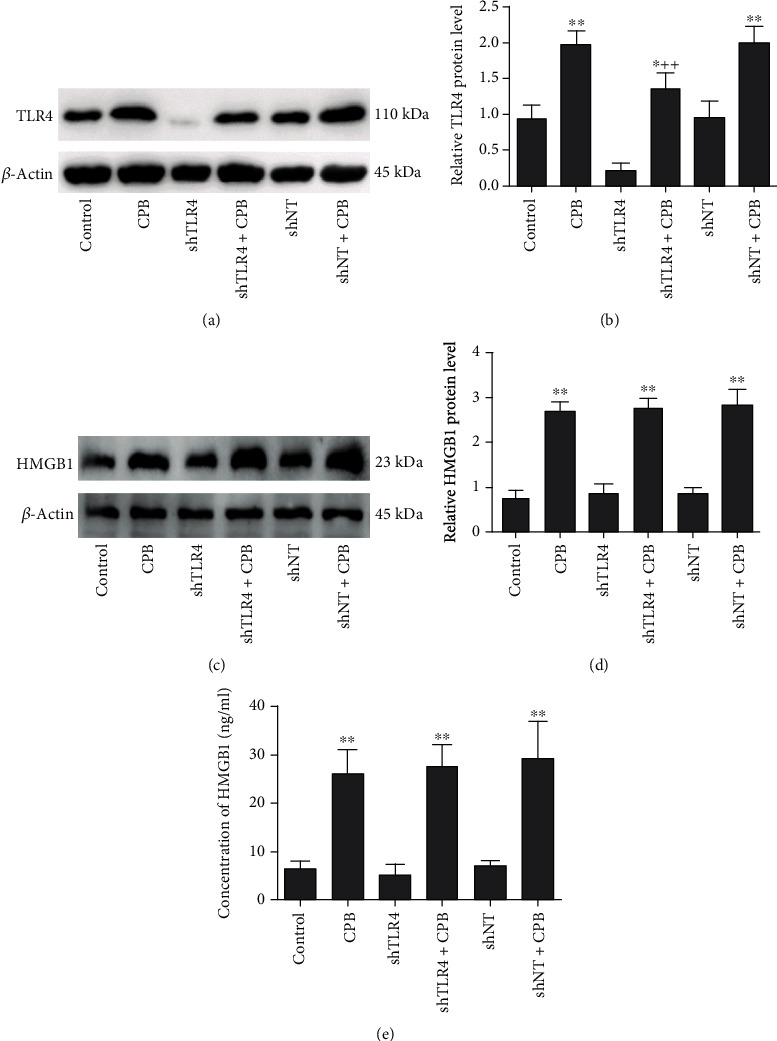
Knocking down TLR4 could not reduce CPB-induced HMGB1 protein expression in the lung tissues. (a) Western blot results showing TLR4 protein expression in the shNT+CPB, shNT, shTLR4+CPB, shTLR4, CPB, and control groups. (b) The TLR4 protein density relative to that of *β*-actin. The TLR4 protein density was significantly lower in the shTLR4+CPB group than in the CPB and shNT+CPB groups (a, b). (c) Western blot results showing HMGB1 protein expression in the shNT+CPB, shNT, shTLR4+CPB, shTLR4, CPB, and control groups. (d) The HMGB1 protein density relative to that of *β*-actin. (e) The concentrations of HMGB1 were evaluated by ELISAs. The reduction of TLR4 by shTLR4 could not reduce the CPB-induced increase in the HMGB1 levels of lung tissues (c–e). Data are represented as the mean ± SD, *n* = 6 per group. ^∗^*P* < 0.05 and ^∗∗^*P* < 0.01 vs. control; ^++^*P* < 0.01 vs. CPB only.

Given that TLR4 functioned downstream of HMGB1, the rats were intratracheally administered lentivirus containing shTLR4 or shNT. When we examined the expression of TLR4 protein in the lung tissues of these rats, we found no difference between the control group and the shNT group (Figures 4(a) and 4(b)). In the lungs of rats in the shTLR4 group without CPB, TLR4 protein expression was 40% of that in the lungs of control rats (Figures 4(a) and 4(b)). Two hours after the termination of CPB, the expression of TLR4 protein in the lung tissue was significantly increased in the CPB-only group and the shNT+CPB group (*P* < 0.01 vs. control) (Figures 4(a) and 4(b)). As expected, the level of TLR4 protein expression in the shTLR4+CPB group was half of that observed in the CPB-only group (*P* < 0.01 vs. CPB only) (Figures 4(a) and 4(b)). To assess whether the TLR4 receptor meditated the CPB-induced HMGB1 protein expression in the lung tissues, we further examined HMGB1 expression in lung tissues by using western blot and ELISA. The HMGB1 levels were significantly higher in the CPB-only, shTLR4+CPB, and shNT+CPB groups than in the control group (*P* < 0.01 vs. control) (Figures 4(c)–4(e)). The results suggest that shTLR4 had no effect on the CPB-induced elevation of HMGB1 levels.

### 3.5. Knocking Down TLR4 Reduced CPB-Induced Acute Lung Injury in Rat Models

**Figure 5:**
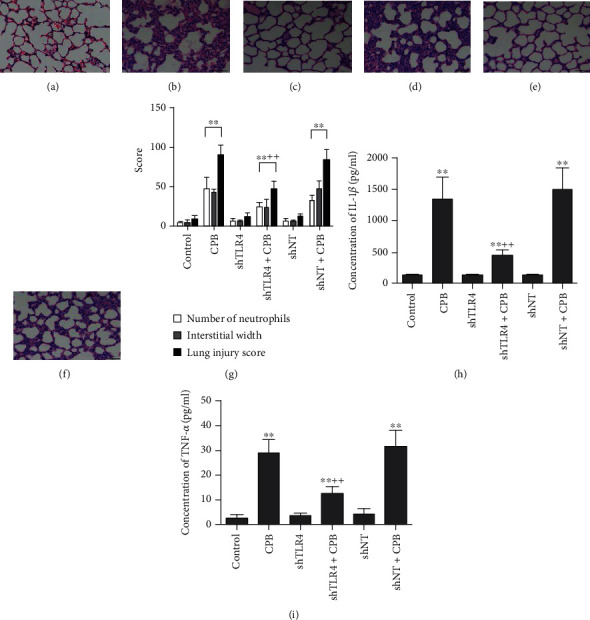
Knocking down TLR4 reduced CPB-induced acute lung injury in rat models. The histological changes of I/R lung injury induced by CPB were evaluated by H&E staining in comparisons of the (a) control vs. (b) CPB, (c) shNT vs. (d) shNT+CPB, and (e) shTLR4 vs. (f) shTLR4+CPB. The number of neutrophils, interstitial width, and lung injury score were quantified and are shown in the different groups (g). Compared with the control group, the CPB-only and shNT+CPB groups showed histologic features of lung injury and increased lung injury scores. In the shTLR4+CPB group, however, lung injury was decreased, and the lung injury scores were lower than those in the CPB-only group. The concentrations of (h) IL-1*β* and (i) TNF-*α* were evaluated by ELISAs. The reduction of TLR4 by shTLR4 attenuated the CPB-induced increase in the IL-1*β* and TNF-*α* levels. Data are represented as the mean ± SD, *n* = 6 per group. ^∗^*P* < 0.05 and ^∗∗^*P* < 0.01 vs. control; ^++^*P* < 0.01 vs. CPB only.

In the presence of successful knockdown of TLR4, we then examined the symptoms of lung injury among these groups. Two hours after the termination of CPB, we observed elevated alveolar septal thickening and vascular congestion and significantly increased interstitial edema and neutrophil infiltration in the lungs of the CPB-only (Figure 5(b)) and shNT+CPB (Figure 5(d)) groups, whereas these features were significantly weaker in the lungs of the shTLR4+CPB group (Figure 5(f)). In addition, the lung injury scores were significantly higher in the CPB-only and shNT+CPB groups than in the shTLR4+CPB group (*P* < 0.01; Figure 5(g)). To determine whether lentiviral infection induces lung injury, we compared the results between the control and shNT groups and observed no difference either in histology (Figures 5(a) and 5(c)) or lung injury scores (Figure 5(g)), indicating that the lung injury was indeed induced by CPB. In the same lung samples described above, we examined the levels of IL-1*β* (Figure 5(h)) and TNF-*α* (Figure 5(i)) by using ELISAs. Similar to anti-HMGB1, shTLR4 could reduce the levels of inflammatory cytokines IL-1*β* and TNF-*α* stimulated upon CPB. However, shNT+CPB showed close levels of IL-1*β* and TNF-*α* as CPB only. Taken together, these data showed that TLR4 functioned downstream of HMGB1 in upregulating IL-1*β* and TNF-*α* after CPB, resulting in severe lung injury phenotypes. Knockdown of TLR4 by intratracheally delivered lentivirus could attenuate CPB-induced lung injury.

## 4. Discussion

In this study, we examined whether the HMGB1-induced activation of the TLR4 signalling pathway is involved in the pathogenesis of lung injury after CPB. We observed lung injuries in a rat model of CPB and found that both the neutralization of HMGB1 by an anti-HMGB1 antibody and the reduction of TLR4 expression via TLR4 shRNA attenuated CPB-induced lung injury. To our knowledge, this is the first study to show that HMGB1-induced TLR4 signalling activation is involved in the pathogenesis of the inflammatory responses in lung tissues after CPB.

Our previous research has found that CPB could induce NLRP3 inflammasome-mediated alveolar macrophage pyroptosis which activates HMGB1 and promotes its release [13]. HMGB1, previously known as a DNA-binding protein, has been shown to induce the release of other cytokines classically associated with ALI including TNF-*α* and IL-1*β* [17]. Actually, the administration of neutralizing antibodies against HMGB1 has been shown to successfully inhibit HMGB1 activity in various animal models and to limit systemic inflammatory response syndrome [18, 19]. In our research, compared with the rats treated with the control IgY antibody, the rats that received the neutralizing anti-HMGB1 antibody had significantly decreased levels of inflammatory cytokines, as well as reduced histologic changes indicative of injury after CPB. These results are consistent with other studies that anti-HMGB1 antibodies could protect against tissue damage caused by different factors, including crush injury leading to the progression to multiple organ failure and haemorrhage-induced brain injury [18, 20]. Recent findings have suggested that targeting the HMGB1-TLR4 signalling cascade may be a novel therapeutic approach for sterile inflammation-related diseases [21–23]. In our study, the expression of TLR4 was also upregulated in the lungs after CPB. According to our team and others previous research showed that HMGB1mediated ALI by upregulating its receptor TLR4 expression [14, 24]. The anti-HMGB1 mAb, as HMGB1 activity neutralizer, could successfully block HMGB1 binding to its downstream receptor TLR4, further inhibiting the upregulation of TLR4 in the lung tissues after CPB. Although the reduction of TLR4 expression with shTLR4 could not inhibit the HMGB1 expression in the lung tissues after CPB, it can decrease CPB-induced levels of inflammatory cytokines in the lungs and attenuate the histologic features of lung injury by blocking the HMGB1 downstream signalling pathway. These results suggested that TLR4 may be involved in HMGB1 leading to CPB-induced ALI. Besides TLR4, HMGB1 has been found to interact with Toll-like receptor 2 (TLR2) and advanced glycation end products (RAGE). However, Li et al. found that HMGB1 acting through RAGE receptor could negatively regulate LPS-mediated tissue damage [25]. In our research, we confirmed HMGB1 as a DAMPs protein contributed to the CPB-related ALI. Thus, RAGE may exert a minor role in HMGB1-mediated ALI induced by CPB. Li et al. found that HMGB1 leads to lung endothelial cell activation, which depends on TLR4 in the early phase and on TLR2 in the late phase following haemorrhagic shock [26]. In our rat model of CPB, the HMGB1 levels in the lungs increased as early as 2 h after the termination of CPB. This result indicated that compared with TLR2, as a HMGB1 receptor, TLR4 plays a major role in the early phase of ALI after CPB. In consideration of TLR4 being the main receptor of HMGB1 during the early stages of ALI induced by CPB, we conclude in our present study that the HMGB1/TLR4 pathway mediated CPB-induced lung injury. Both the cyclic stretch and hepatic ischemia-reperfusion-induced HMGB1 expression were mediated through NF-*κ*B signalling pathways [27, 28]. Other research has confirmed that pulmonary I/R injury can lead to NF-*κ*B activation [29, 30]. Notably, TLR4 directly activates NF-*κ*B signalling pathways and the expression of proinflammatory cytokines such as IL-1*β* and TNF-*α* [31]. Thus, we can speculate that CPB can activate the HMGB1/TLR4/NF-*κ*B signalling pathways, which lead to ALI and promote cytokine expression.

According to our experimental research, neither anti-HMGB1 nor shTLR4 influenced the mortality rate (data not shown) or aggravated lung injury in normal rats (Figures 3 and 5), which imply the safety of the treatment of anti-HMGB1 antibody and shTLR4 in animal experiment. Anti-HMGB1 strategy [32] and viral vectors [33] were also reported to be safe for clinical therapy. Therefore, it is reasonable to speculate that the treatment of anti-HMGB1 antibody or shTLR4 may be safe for future clinical treatment in CPB-induced ALI. In spite of shTLR4 application showing more negative effects in inflammatory factor release after CPB in our present study, current clinical experimental studies showed no statistical significance in alleviating the tissue damage and inflammatory factor expression by ethyl pyruvate (EP, the HMGB1 inhibitor) or TAK-242 (TLR4 antagonist) treatment in CPB [34] or sepsis-induced inflammation [35], so the clinical effects of the TLR4/HMGB1 pathway inhibition strategy need to be further evaluated in the future study.

## 5. Conclusions

In conclusion, the data presented in this study indicate that CPB induced ALI through the HMGB1/TLR4 pathway, and inhibition of either TLR4 or HMGB1 expression in the lung tissues can reduce CPB-induced ALI.

## Data Availability

All datasets are included in the manuscript.
